# Increased Time Difference between Imagined and Physical Walking in Older Adults at a High Risk of Falling

**DOI:** 10.3390/brainsci10060332

**Published:** 2020-05-29

**Authors:** Hideki Nakano, Shin Murata, Kayoko Shiraiwa, Koji Nonaka

**Affiliations:** 1 Department of Physical Therapy, Faculty of Health Sciences, Kyoto Tachibana University, 34 Yamada-cho, Oyake, Yamashina-ku, Kyoto-city, Kyoto 607-8175, Japan; murata-s@tachibana-u.ac.jp (S.M.); shiraiwa@tachibana-u.ac.jp (K.S.); 2 Department of Rehabilitation, Faculty of Health Sciences, Naragakuen University, 3-15-1 Nakatomigaoka, Nara-city, Nara 631-8524, Japan; nonaka@naragakuen-u.jp

**Keywords:** mental chronometry, motor imagery, older adult, risk of fall, walking

## Abstract

Walking motor imagery ability is thought to be associated with a fear of falling; however, no studies have compared fall risk and motor imagery ability. This study aimed to ascertain the time difference between imagined and physical walking in older adults at low and high risks of falling. Motor imagery ability was assessed using mental chronometry, which measures the imagined time required for movement. Participants included 31 older adults classified as having a high (*n* = 15) or low (*n* = 16) risk of falling based on single leg stance time. The time required for imagined and physical walking was measured using 5 m long walkways with three different widths (15, 25, and 50 cm), and the temporal errors (absolute and constant error) were compared. Physical walking time was significantly longer in the high-risk group than in the low-risk group for the 15 and 25 cm wide walkways. The absolute error between the imagined and physical walking times was significantly larger in the high-risk group than in the low-risk group for the 15 and 25 cm wide walkways. There was also a significant difference in the constant error between the high- and low-risk groups between the imagined and physical walking times for all three walkways. Older adults who may be at a higher risk of falling showed longer walking times during action execution but overestimated their performance (i.e., they believe they would be faster) during motor imagery. Therefore, the time difference between imagined and physical walking could, in part, be useful as a tool for assessing fall risk based on motor imagery.

## 1. Introduction

Motor imagery has been studied from various perspectives such as simulation theory [1], emulation theory [2], and the moto-cognitive model [3]. In simulation theory, motor imagery is defined as mentally simulating a movement without actually moving [1]. A previous study reported that motor imagery involves the offline activity of many motor areas, such as premotor areas, supplementary motor areas, and the cerebellum [4]. Moreover, according to a recent meta-analysis, motor imagery activates the frontal–parietal network in addition to the basal ganglia, cerebellum, and other regions of the brain that are activated during actual movement [5]. These are the defining features of simulation theory—specifically, that motor imagery is the psychological counterpart of the offline operation of the efferent motor areas [2].

Mental chronometry is one of the tools used to assess motor imagery ability [6,7,8,9]. Mental chronometry measures the imagined time required for movement. Smaller differences between imagined and actual times are suggestive of a higher motor imagery ability. The motor imagery ability of walking is known to change with age [10]. Previous studies have shown that the temporal error between imagined walking and physical walking is higher in older adults and frail older adults than in younger people due to the age-related decline in working memory [11,12,13,14]. It has also been reported that the increased level of temporal error between imagined and physical walking in older adults is related to a decline in motor function and a greater fear of falling [15,16,17]. These findings suggest that the motor imagery ability of walking in older adults declines with age and may be related to a high risk of falling—and therefore, actual falling—but this is yet to be determined. Clarifying this information may contribute to the evaluation of the risk of falling in older adults based on the motor imagery ability of walking. 

Therefore, the aim of this study was to determine the temporal characteristics of imagined walking and physical walking in older adults with different risk levels of falling. According to a previous study, an increased time difference between imagined and physical walking (longer imagined walking time compared to physical walking time) was observed in older adults with a fear of falling [17]. Hence, a similar phenomenon is expected in older adults with a high risk of falling, as there is an association between risk and fear of falling [18]. Moreover, another study reported that the prefrontal cortex, which is involved in the cognitive states of simulation theory, is involved in the evaluation of one’s own ability [19]. Therefore, a large time difference between imagined walking and actual walking in elderly people who have a high risk of falling could imply that prefrontal cortex impairment is present.

This study is the first to investigate the temporal characteristics of imagined and physical walking in older adults with high and low risks of falling.

## 2. Materials and Methods

### 2.1. Participants

The population from which the study sample was drawn included 57 older adults using long-term care insurance services in Japan [20]. Older adults who use long-term care insurance services have physical or mental disorders and need support to perform activities of daily living [21]. We excluded 20 participants with Mini-Mental State Examination (MMSE) [22] scores below 24; those with orthopedic, neurological, or psychiatric diseases that may have affected the outcomes of this study; and those who were unable to walk independently, as evaluated by the physical therapist in charge of the participants. Finally, 31 older adults (men *n* = 17, women *n* = 14, mean age 79.29 ± 5.96 years, mean height 154.79 ± 12.96 cm, mean weight 59.28 ± 10.91 kg, mean MMSE score 27.45 ± 2.14) were included. The sample size was calculated using G * Power [23,24].

This study was conducted in accordance with the tenets of the 1975 Declaration of Helsinki, which was revised in 2013. Signed written consent was obtained from all participants following a full explanation of the aim and content of the study, the benefits and risks, and the protection of personal information and information regarding the refusal or withdrawal of consent. The study was approved by the Kyoto Tachibana University Research Ethics Committee.

### 2.2. Measurement

First, single leg stance (SLS) time was measured using a digital stopwatch for all participants with their eyes open [25,26]. Participants were instructed to place both arms beside their bodies and gaze at a mark 2 m away; the time from foot lift to touching the floor was measured. Measurements were taken twice for each foot, and the longest time (seconds) was used for analysis. Previous studies have reported that single leg standing balance (the ability to stand on one leg for 5 s without assistance) is a predictor of injurious falls in older adults [26,27]. In this study, participants with an SLS time of less than 5 s were classified as belonging to the high-risk fall group (men *n* = 7, women *n* = 8, mean age 79.13 ± 6.08 years, mean height 156.77 ± 59.34 cm, mean weight 59.34 ± 12.94 kg, mean MMSE score 27.60 ± 1.96, SLS time 2.36 ± 1.47 s), and participants with an SLS time of 5 s or longer were classified as belonging to the low-risk fall group (men *n* = 10, women *n* = 6, mean age 79.44 ± 6.04 years, mean height 152.94 ± 15.36 cm, mean weight 59.23 ± 9.05 kg, mean MMSE score 27.31 ± 2.36, mean SLS time 18.62 ± 15.33 s).

Next, motor imagery ability was assessed using mental chronometry [6,7,8,9]. We measured the time taken for imagined walking and physical walking using walkways of three different widths (50 cm, 25 cm, and 15 cm wide × 5 m long) [6,7]. The measurement methods were implemented similarly to previous studies [13,14]. First, the participant stood in front of one of the three walkways and completed the task of imagining walking along that walkway. Mental chronometry was used to measure the imagined walking, and the participants used a stopwatch to indicate the start and end of the imagination. Next, the same task was implemented for the remaining two walkways. The order of the walkways was randomized. Following imagined walking, the participants stood in front of one of the three walkways and completed the physical walking trials along the three walkways. The participants were instructed to walk (using their imagination) at a normal speed. The experimenters used a stopwatch to measure the walking speed. Subsequently, the same task was implemented for the remaining two walkways. Once again, the order of the measured walkways was selected at random. Each measurement was performed twice, and the second value was used for analysis.

The temporal error (absolute error and constant error) between imagined walking and physical walking was calculated from the obtained data using the following equations [11,12,15,28]:

Absolute error = |Executed Time − Imagined Time/(Executed Time − Imagined Time)/2|(1)

Constant error = Executed Time − Imagined Time/(Executed Time − Imagined Time)/2.(2)

Constant error was used to examine the bias between actual and imagined times. This score reflects a participant’s bias in performance. However, negative and positive values can cancel each other out when averaging. Therefore, it is important to report an error term that is not affected by scores of opposing signs. In this context, the absolute error provided a measure that was independent of directional bias and reflected the overall accuracy [11]. The difference in executed minus imagined times was calculated for each participant and then averaged across each group.

### 2.3. Statistics Analysis 

The Kolmogorov–Smirnov test was used to check normality; parametric tests were selected for normally distributed variables, and non-parametric tests were selected for non-normally distributed variables. The Student’s *t*-test was used to compare age, height, weight, and MMSE score; the Mann–Whitney U test was used to compare the SLS time between the high- and low-risk groups. A repeated measures analysis of variance (ANOVA) was used to compare the imagined and physical walking times on the three walkways between the high- and low-risk groups, and Bonferroni-corrected *t*-tests were used for post-hoc analysis. In addition, a repeated-measures ANOVA was used to compare the temporal error (absolute error and constant error) between imagined and physical walking times, and Bonferroni-corrected *t*-tests were used for post-hoc comparisons. SPSS 24.0 was used for statistical analysis, and the significance level was set at 0.05.

## 3. Results

A repeated-measures ANOVA with the between-participant factor risk (low risk, high risk) and the within-participant factors of action (imagination, execution) and walkway width (50 cm, 25 cm, 15 cm) was performed on the durations. In addition, a repeated-measures ANOVA with the between-participant factor risk (low risk, high risk) and walkway width (50 cm, 25 cm, 15 cm) was performed on the absolute and constant errors. 

**Figure 1:**
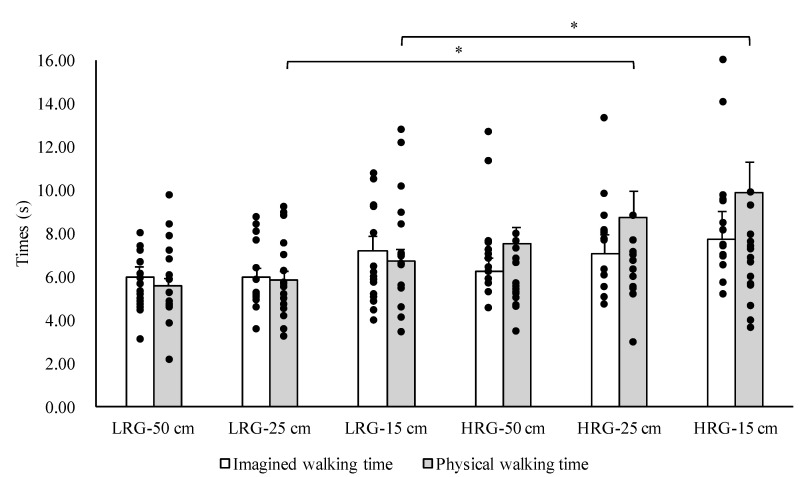
Comparison of the imagined and physical walking times of the high-risk group (HRG) and low-risk group (LRG) for each of the three walkway widths. Data are shown as the mean ± standard error. * *p* < 0.05.

**Figure 2:**
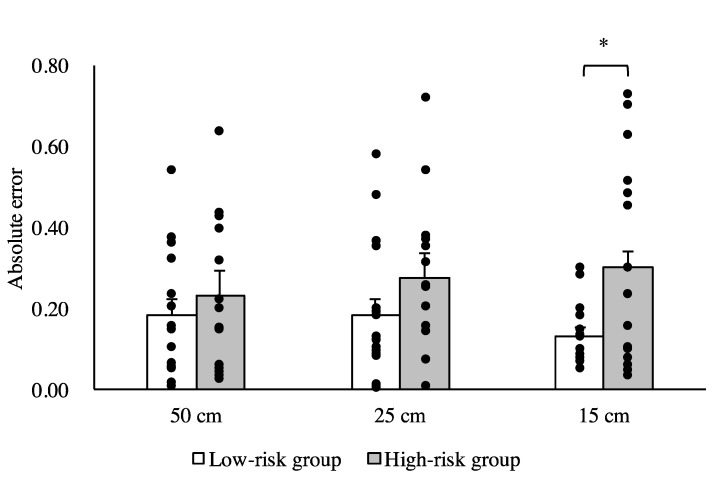
Comparison of the absolute error between the high- and low-risk groups for each of the three walkway widths. Data are presented as the mean ± standard error. * *p* < 0.05.

**Figure 3:**
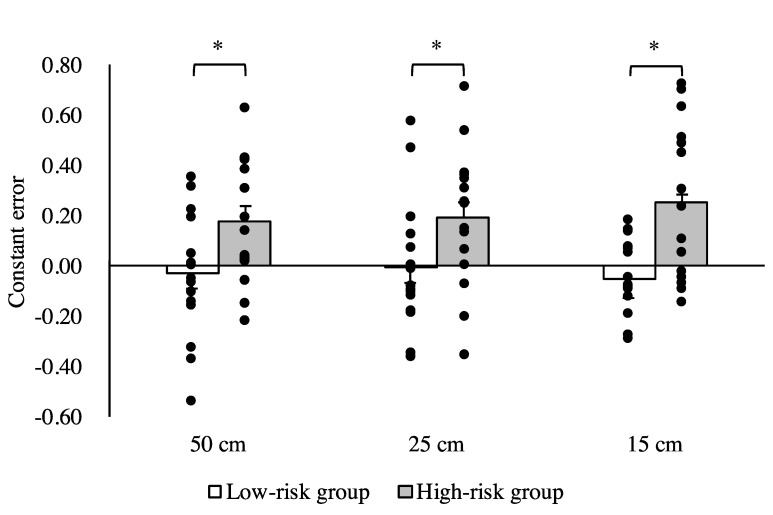
Comparison of constant error between the high- and low-risk fall groups for each of the three walkway widths. Data are presented as the mean ± standard error. * *p* < 0.05.

No significant differences between the low- and high-risk groups in terms of age, height, weight, or MMSE score (*p* > 0.05) were observed. There was a significant difference between the high- and low-risk groups in SLS time (*p* < 0.05). The results of the three-way ANOVA revealed significant main effects of the group and width factors (F = 11.94, *p* < 0.05; F = 3.46, *p* < 0.05, respectively) and a significant interaction between group and condition (F = 4.37, *p* < 0.05). The results of the post-hoc test revealed that physical walking times for the 25 and 15 cm widths were significantly greater in the high-risk group compared with the low-risk group (Figure 1). The results of the two-way ANOVA revealed a significant main effect of group on the absolute error (F = 11.40, *p* < 0.05). The results of the post-hoc analysis revealed that the absolute error was significantly greater in the high-risk group for the 25 and 15 cm wide walkways compared with the low-risk group (*p* < 0.05) (Figure 2). Similarly, there was a significant main effect of group on the constant error (F = 26.74, *p* < 0.05). The results of the post-hoc test revealed significant differences in the constant error between the high- and low-risk groups for the 50, 25, and 15 cm wide walkways (*p* < 0.05) (Figure 3). 

The effect sizes of the between-group differences in the absolute and constant errors were calculated using Cohen’s d [29]. The effect sizes of absolute error were as follows: 50 cm, 0.29 (small); 25 cm, 0.53 (medium); 15 cm, 0.95 (large). Moreover, the effect sizes of constant error were as follows: 50 cm, 0.88 (large); 25 cm, 0.76 (medium); 15 cm, 1.31 (large).

## 4. Discussion

In this study, the physical walking time in the high-risk group was significantly longer than tht in the low-risk group for the 25 and 15 cm wide walkways. The temporal error (absolute error) between the imagined and physical walking times for the high-risk group was significantly greater than that for the low-risk group on the 25 and 15 cm wide walkways. Furthermore, there was a significant difference in temporal error (constant error) between the high- and low-risk groups between the imagined and physical walking times on all three walkways. Specifically, the constant error for the low-risk group was either close to zero or negative (the imagined walking time was longer than the physical walking time), while the high-risk group had positive values for constant error (the imagined walking time was shorter than the physical walking time). These results show that older adults who are possibly at a higher risk of falling have longer walking times during action execution but overestimate their performance (i.e., they believe they will be faster) during motor imagery.

Previous studies have clarified that the temporal error between imagined and physical walking is larger in (frail) older adults than in (healthy) younger adults [11,12,13,14,30]. It has also been reported that temporal error is greater in older adults with a fear of falling than in those without a fear of falling [16,17]. A similar phenomenon was observed in this study; temporal error was higher in older adults with a high-risk of falling compared with older adults with a low risk of falling. We measured the imagined walking time using mental chronometry, which reflects the motor imagery ability; smaller differences between imagined and actual times reflect a higher level of motor imagery ability, and larger differences indicate lower levels of motor imagery ability [6,7,8,9]. Therefore, the abovementioned results suggest that motor imagery ability is lower in older adults with a high risk of falling compared with older adults with a low risk of falling.

Grenier et al. found that the temporal error between imagined and physical walking was significantly greater in older adults with a fear of falling than in older adults without a fear of falling, and they also found a significant correlation between temporal error and fear of falling [16]. A similar study by Sakurai et al. reported that older adults with a fear of falling underestimated their walking times under imagined walking conditions (i.e., overestimation of their own walking ability) [17]. It has been clarified that the reason for these characteristics is that activity avoidance due to a fear of falling and an inactive lifestyle causes a decline in the motor imagery ability of older adults [31]. It is also known that older adults with a fear of falling have a reduction in brain metabolic activity in the supplementary motor area, which is responsible for motor imagery [32]. We did not evaluate fear of falling in the older adults who participated in the study, but given that there is an association between risk of falling and fear of falling [18], it was assumed that the high-risk group had a greater fear of falling. The above findings suggest that the increased temporal error (absolute error and constant error) between imagined and physical walking in the high-risk group may have been caused by a greater fear of falling. Further, we suggest that an inactive lifestyle (due to fear of falling) reduced motor image ability, causing increased levels of temporal error between imagined and physical walking times in the high-risk group.

The high-risk group had positive values for constant error (imagined walking time was shorter than physical walking time), whereas the constant error for the low-risk group was either close to zero or negative (imagined walking time was longer than physical walking time). These results suggest that the-high risk group overestimated their walking ability more than the low-risk group. A previous study reported that elderly fallers overestimated their step-over ability more than elderly non-fallers [33]. Moreover, a longitudinal study showed that overestimation of step-out ability is a good predictor of future falls [34]. Collectively, this suggests that the estimation error (overestimation) of one’s own walking ability, which is calculated from the time difference between the real and the imagined walking tasks, is an additional explanation for the high risk of falls in the elderly.

In addition to the above, previous studies have reported that neural changes in the anterior prefrontal cortex, especially in the orbitofrontal cortex, negatively influence a person’s ability to properly evaluate their ability [19,35,36]. In simulation theory, action representations can operate offline via a simulation mechanism, as the motor system is part of a cognitive network [4,37]. Especially, the orbitofrontal cortex exerts an inhibitory influence on areas involved in cognitive states to simulated actions [4]. Hence, this suggests that the high-risk group may not be able to properly perform motor imagery due to functional decline in the orbitofrontal cortex.

The present study has some limitations. First, the participants’ fear of falling was not measured. Therefore, clarification of the association between risk and fear of falling and the temporal error between imagined and physical walking in older adults need to be examined in future studies. Second, we did not conduct a detailed evaluation of the motor and cognitive functions of the participants. Future studies are needed to investigate the association between temporal error between imagined and physical walking and motor and cognitive function in older adults. Third, imagined walking time was measured by the participants, and physical walking time was measured by the experimenters. Therefore, it is possible that some biases and differences between the conditions resulted from this methodological difference, and the results may not properly reflect motor imagery and walking ability. It is necessary to unify the measurement methods in future studies and verify the results using a reliable and valid measurement method. Fourth, the participants were only instructed to walk at a normal speed for both the imagined and execution conditions. However, changing the walking speed may make the participant’s motor imagery more visible. Therefore, it is necessary to examine changes in motor imagery ability due to differences in walking speed in future studies.

## 5. Conclusions

This study showed that the temporal error (absolute error) between the imagined and physical walking times for the high-risk group was significantly greater than for the low-risk group. Furthermore, there was also a significant difference between the high- and low-risk groups for the temporal error (constant error) between the imagined and physical walking times. These findings suggest that increased temporal error (absolute error and constant error) between imagined and physical walking in the high-risk group may have been caused by a greater fear of falling. Therefore, we suggest that the time difference between imagined and physical walking could, in part, be useful as a tool for assessing fall risk based on motor imagery.
